# Internal Ribosome Entry Site Dramatically Reduces Transgene Expression in Hematopoietic Cells in a Position-Dependent Manner

**DOI:** 10.3390/v11100920

**Published:** 2019-10-08

**Authors:** Qingyun Zheng, Xueyan Zhang, Hua Yang, Jinyan Xie, Yilin Xie, Jinzhong Chen, Chenghui Yu, Chen Zhong

**Affiliations:** 1 State Key Laboratory of Genetic Engineering, School of Life Sciences, Fudan University, Shanghai 200438, China; 18210700156@fudan.edu.cn (Q.Z.); 15307110317@fudan.edu.cn (X.Z.); 18210700046@fudan.edu.cn (J.X.); 15307110332@fudan.edu.cn (Y.X.); kingbellchen@fudan.edu.cn (J.C.); 2 Division of Cellular and Molecular Therapy, Department of Pediatrics, University of Florida College of Medicine, Gainesville, FL 32610, USA; h.yang@ufl.edu; 3 Department of Radiology, Central South University, Changsha, Hunan 410013, China; 4 Yeda Research Institute of Gene and Cell Therapy, Taizhou, Zhejiang 318000, China

**Keywords:** internal ribosome entry site (IRES), recombinant adeno-associated virus vector, hematopoietic cells, bicistronic transgene, gene therapy

## Abstract

Bicistronic transgene expression mediated by internal ribosome entry site (IRES) elements has been widely used. It co-expresses heterologous transgene products from a message RNA driven by a single promoter. Hematologic gene delivery is a promising treatment for both inherited and acquired diseases. A combined strategy was recently documented for potential genome editing in hematopoietic cells. A transduction efficiency exceeding ~90% can be achieved by capsid-optimized recombinant adeno-associated virus serotype 6 (rAAV6) vectors. In this study, to deliver an encephalomyocarditis virus (EMCV) IRES-containing rAAV6 genome into hematopoietic cells, we observed that EMCV IRES almost completely shut down the transgene expression during the process of mRNA–protein transition. In addition, position-dependent behavior was observed, in which only the EMCV IRES element located between a promoter and the transgenes had an inhibitory effect. Although further studies are warranted to evaluate the involvement of cellular translation machinery, our results propose the use of specific IRES elements or an alternative strategy, such as the 2A system, to achieve bicistronic transgene expression in hematopoietic cells.

## 1. Introduction

Bicistronic transgene expression is currently essential in gene therapy and biomedical research. The application of internal ribosome entry site (IRES) elements can co-express dual heterologous transgene products from a message RNA driven by a single promoter [1,2]. Generally, translation in eukaryotes begins at the 5’ end cap of the mRNA molecule, where translation initiation factors are recruited [3,4,5,6]. On the other hand, IRES elements that mimic the 5’ cap structure allow for translation in an RNA cap-independent manner [7,8]. The process is assisted by diverse RNA binding proteins and ribosomal subunits [9,10,11]. Trans-acting factors vary with distinct IRES elements, resulting in different translational efficiencies based on cell types and cellular conditions [12]. One type of IRES element that is derived from encephalomyocarditis virus (EMCV) has been widely used for pharmaceutical and biomedical applications. It initiates a higher translation efficiency than other viral and non-viral IRES elements [13,14]. Nevertheless, it is well accepted that the efficiency of the IRES-governed downstream open reading frame (ORF) translation is lower than that of the cap structure-governed upstream ORF translation. In most cases, it is between 5% and 50%, regardless of transgene delivery methods [15]. 

Gene delivery in hematopoietic stem/progenitor cells (HSPCs), T and B lymphocytes is promising for the treatment of both inherited and acquired diseases. Many approaches using viral vectors have been considered to achieve gene therapy for hematologic diseases [16,17,18,19,20]. However, the widespread clinical use of these vectors has long been hampered by limitations in efficacy and safety. For example, in phase I/II clinical trials, genetic modification was achieved in only 9–14% of blood cells after transplant of a self-inactivating lentiviral vector [21]. Although in the laboratory nearly 100% expression of GFP transgenes in human CD34+ cells can be achieved in vitro [22,23,24], the lentiviral vectors are considered to potentially induce cancer by the dysregulation of cell growth, mutagenesis, and reorganization. This is one of the greatest challenges for hematologic gene therapy clinical trials.

Based on a nonpathogenic parvovirus, the recombinant adeno-associated virus (rAAV) vector has been developed as a gene therapy drug [25]. Initially, Song, et al. evaluated the transduction efficiency of all available rAAV serotype vectors (rAAV1-rAAV10) and observed that rAAV6 was the most efficient in human HSPCs [26]. In addition, the transduction efficiency can be further improved by specifically mutating surface-exposed tyrosine residues to phenylalanine (Y445F, Y705F or Y731F) on the rAAV6 capsid [27]. Based on these findings, a number of research groups have achieved efficient gene editing with the help of rAAV6 vectors in human hematopoietic cells [28,29,30]. Most recently, a combined strategy was documented for potential genome editing in hematopoietic cells, with which a transduction efficiency exceeding ~90% can be achieved by a capsid-optimized rAAV6 vector. In the present study, based on the capsid-optimized rAAV6 vectors, we characterized the inhibitory effect of the EMCV IRES element on the downstream transgene expression in hematopoietic cells. Our results could contribute to broadening the understanding of IRES-mediated transgene inhibition in hematopoietic cells and provide an optimal strategy to co-express dual genes using capsid-optimized rAAV6 vectors for potential gene therapy.

## 2. Materials and Methods

### 2.1. Cell Culture

The human embryonic kidney cell line HEK293, hematopoietic cell lines K562, Jurkat and THP-1, as well as cervical epithelial carcinoma cell line HeLa were purchased from the American Type Culture Collection (Manassas, VA, USA). Human embryonic kidney cell line 293T was purchased from the Institute of Biochemistry Cell Biology (Shanghai, China). The CD34+ hematopoietic stem cells (HSCs) were purchased from ALLCELLS (Alameda, CA, USA). The hepatocellular carcinoma cell line Huh7 was obtained from Dr. Chen Liu’s laboratory at the Cancer Institute of New Jersey Rutgers Health. HEK293, HEK293T, Huh7, and HeLa cells were cultured in complete Dulbecco’s Modified Eagle Medium (DMEM) supplemented with 10% fetal bovine serum and 1% penicillin–streptomycin. K562 cells were maintained in complete Iscove’s-modified Dulbecco’s medium (IMDM) as a suspension culture. Jurkat and THP-1 cells were maintained in complete Roswell Park Memorial Institute (RPMI)-1640 medium as a suspension culture. CD34+ HSCs were cultured in complete StemSpan’s Serum-Free Medium (SFEM) for Expansion with 1% StemSpan™ CC100 (STEMCELL Technologies, Vancouver, BC, Canada), which supports the proliferation of human hematopoietic cells. 

To isolate CD4+ T cells, peripheral blood mononuclear cells (PBMCs) were isolated from whole blood by density gradient centrifugation using Lympholyte®-H Cell Separation Media (Cedarlane Laboratories, Burlington, ON, Canada). CD4+ T cells were purified from PBMCs by negative selection using a CD4+ T cell Isolation Kit (Miltenyi Biotech, Bergisch Gladbach, Germany). The primary CD4+ T cells were then stimulated with Dynabeads® Human T-Activator CD3/CD28 (Life Technologies, Thermo, Waltham, MA, USA) at a ratio of 1:1 for 3 days and cultured in RPMI-1640 medium (Gibco, Thermo, Waltham, MA, USA) supplemented with 10% fetal bovine serum (Gibco) and 5 ng/mL of recombinant human IL-2 (R&D systems, Minneapolis, MN, USA). All cells were grown in a cell incubator at 37 °C in an atmosphere containing 5% CO_2_. 

### 2.2. Plasmid Transduction

Polyethylenimine (PEI) and Lipofectamine 2000 were purchased from Sigma-Aldrich (St. Louis, MO, USA) and Invitrogen (Thermo, Waltham, MA, USA), respectively. Electrotransformation was performed using 1 × 10^6^ K562 cells/reaction and 5 μg of plasmids (pAAV-CMVp-*gfp*, 1000 ng/μL). The cells were resuspended in electroporation buffer with specific plasmids (pAAV-CMVp-*gfp*, 1000 ng/μL), and the suspensions were transferred into cuvettes and electroporated at 360 V for 30 ms using an electroporator (Celetrix, Manassas, VA, USA). K562 cells were transferred to a six-well plate and cultured in complete culture medium immediately after electroporation. Flow cytometry was carried out at 72 hours post-transduction.

### 2.3. AAV Vector Production

AAV was packaged by a PEI-mediated triple-plasmid transfection method [31]. The plasmids of the gene of interest, capsid protein plasmid (pACG2-C6-3M, which was mutated at three surface-exposed amino acids including T492V, Y705F, and Y731F), and pHelper plasmid were simultaneously transferred into the HEK293 cells. At 72 hours after transfection, HEK293 cells were collected and ruptured by repeated freezing and thawing. The purification methods of the viral vectors included density gradient centrifugation in iodixacol solution and filtration in a HiTrap Q HP ion-exchange column. Quantitative real-time PCR (qRT-PCR) was performed using the 2xT5 SYBR Green Fast qPCR Mix kit (TSE202, Tsingke Biological Technology, Beijing, China) to determine the virus titer. 

### 2.4. AAV Vector Transduction

Transduction assays were performed as previously described [32,33]. Briefly, adherent cells (HEK293, Huh7 and HeLa), suspension cells (K562, THP-1 and Jurkat), as well as primary CD34+ HSCs and CD4+ T cells were transduced with purified AAV vectors at an MOI of 10,000 vgs/cell. All the AAV transductions were carried out in FBS-free medium for 2 hours. Cells were then switched to FBS-containing medium for growth. At 72 hours post-infection, the transgene delivery efficiency was quantified by the percentage of GFP-positive or Fluc-positive cells using flow cytometry or firefly luciferase assay. Alternatively, GFP expression was analyzed under fluorescence microscopy and quantitated by Image J software (National Institutes of Health, Bethesda, MD, USA).

### 2.5. Lentiviral Production and Infection

Lentivirus (LV) was produced by transfecting 293T cells with a plasmid (pLV-EMCV IRES-*gfp*) encoding the lentivirus and two packaging plasmids (pMD2.G and psPAX2) [32]. The supernatant harboring the lentivirus particles was harvested at 48 hours post-transduction. Subsequently, HEK293 and K562 cells were infected with the supernatant and incubated for 6 hours with 8 mg/mL of hexadimethrine bromide (Polybrene, Sigma-Aldrich, St. Louis, MO, USA) before reverting to normal medium. The transgene expression was detected by flow cytometry at 7 days post-infection, indicating the percentage of GFP-positive cells.

### 2.6. GFP Determination 

Total cellular DNA was isolated using a Beyotime kit (D0063, Beyotime Biotechnology, Shanghai, China). Trypsin was used while harvesting cells to remove virus vectors attached to the cell surfaces. Total RNA was isolated using a Takara kit (CAT#9767, Takara Bio Inc., Shiga, Japan). cDNA was generated by reverse transcription (RR036A, Takara Bio Inc, Shiga, Japan) of the total RNA. Furthermore, 100 ng of DNA and cDNA samples were subjected to qPCR using 2 x T5 SYBR Green Fast qPCR Mix (TSE202, Tsingke Biological Technology, Beijing, China). The forward primer was 5’ GTGGTGTACATGAACGACGG, and the reverse primer was 5’ CCACGTAGGTCTTCTCCAGG. 

For the adherent cell, fluorescence microscopy and Image J analysis software were used to detect and quantitatively analyze the GFP expression. For the suspension cells, flow cytometry using a Calibur Flow Cytometer (BD Biosciences, San Diego, CA, USA) was performed to quantitatively determine the GFP expression. The cells were harvested, rinsed, resuspended in PBS, and analyzed with the FL-1 channel. FlowJo software (FlowJo, LLC, Ashland, OR, USA) was used to calculate the percentage of fluorescent cells in different groups.

### 2.7. Western Blot Analysis

Western blot assays were performed as previously described [33]. HEK293 and K562 cells were harvested, rinsed by PBS, and treated with cell lysis buffer (P0013B, Beyotime Biotechnology, Shanghai, China). The amount of total protein was measured using a BCA Protein Assay Kit (P0012, Beyotime Biotechnology, Shanghai, China). Quantified protein samples were separated by SDS-PAGE and transferred to poly-vinylidene difluoride transfer (PVDF) membranes. The membranes were blocked by nonfat milk at room temperature for 2 hours, followed by incubation with Anti-Gemin5 primary antibody (1:200, sc-136200, Santa Cruz Biotechnology, Santa Cruz, CA, USA), Anti-PTBP1 (1:500, 32-4800, Thermo, Waltham, MA, USA), Anti-PCBP2 (1:1000, PA5-30116, Thermo, Waltham, MA, USA), Anti-GAPDH primary antibody (1:1000, AF0006, Beyotime Biotechnology, Shanghai, China), Anti-Rabbit secondary antibody (1:1000, A0208, Beyotime Biotechnology, Shanghai, China), and Anti-Mouse secondary antibody (1:1000, A0216, Beyotime Biotechnology, Shanghai, China). An ECL kit was used to visualize the protein expression (Cat#180-501, Tanon, Shanghai, China).

### 2.8. Firefly Luciferase Assay

Firefly luciferase detection was performed with a Firefly Luciferase Reporter Gene Assay Kit (RG005, Beyotime Biotechnology, Shanghai, China). Transduced cells were collected after 3 days, rinsed with PBS, and lysed in cell lysis buffer in an ice bath for 30 min. The liquid supernatant was collected after centrifugation at 3500 rcf for 15 min. An equivalent amount of luciferase detection reagent was added to the samples, and the chemiluminescence was detected in a multifunctional enzyme-labeling apparatus (Synergy™ 2, BioTek, Winooski, VT, USA).

### 2.9. Statistical Analysis

All experiments were performed in triplicate at least. The software GraphPad Prism 5.0 (GraphPad, San Diego, CA, USA) was used for statistical analyses of the data, which are shown as the mean ± the standard deviation (S.D). Differences between two groups were compared using the nonparametric Mann–Whitney U test; a one-way ANOVA followed by Dunnett’s test was used in the case of three or more groups. A *p* value < 0.05 was considered as significantly different: *p* < 0.05 (*), *p* < 0.01 (**), *p* < 0.001 (***). 

## 3. Results

### 3.1. Capsid-Optimized rAAV6 Vector Mediated Efficient Transduction in Hematopoietic Cells

**Figure 1:**
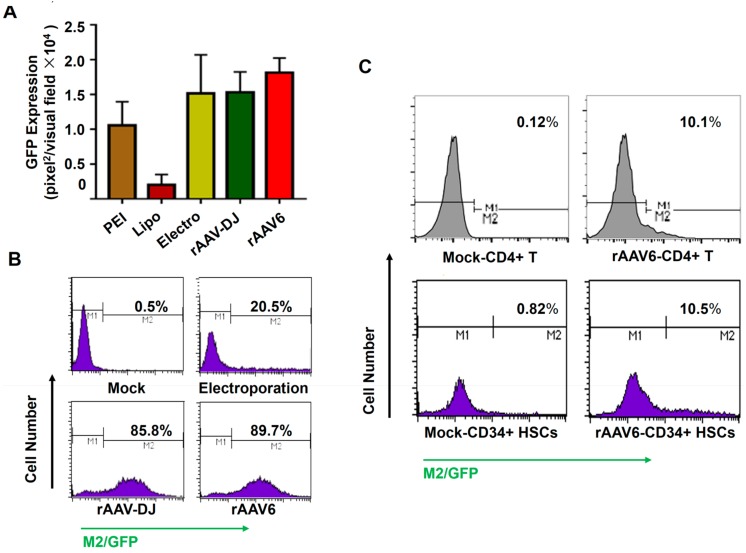
Capsid-optimized recombinant adeno-associated virus serotype 6 (rAAV6) vectors represented the most efficient gene delivery method for hematopoietic cells. (**A**) K562 cells were transduced with the *gfp* gene through various indicated methods. Transgene expression was detected by fluorescence microscopy at 72 hours post-transfection or post-viral transduction. (**B**) Transgene expression from (**A**) was measured by flow cytometry. (**C**) Primary human CD4+ T cells and CD34+ hematopoietic stem cells (HSCs) were transduced with rAAV6-CMVp-*gfp* vectors at 10,000 vgs/cell. Transgene expression was detected by flow cytometry at 72 hours post-transduction. PEI: polyethylenimine.

Various known high-efficiency transgene delivery strategies were explored to deliver the *gfp* gene in K562 cells, including polyethylenimine, lipofectamine, electro-transfection, rAAV-DJ, and capsid-optimized rAAV6 vectors. As shown in Figure 1A, electro-transfection, rAAV-DJ, and capsid-optimized rAAV6 vectors led to higher GFP expression, which were determined by fluorescent microscopy. Further characterization by flow cytometry revealed that electro-transfection resulted in a lower GFP-positive percentage of cells with higher transgene expression in each GFP-positive cell (Figure 1B). The capsid-optimized rAAV6 vectors had a slightly higher transduction efficiency than rAAV-DJ vectors. In addition, the capsid-optimized rAAV6 vectors conferred higher resistance to pooled intravenous immunoglobulin (IVIG) neutralization in comparison to their wild-type (WT) counterparts (data not shown) [34]. IVIG at 1 mg/mL was able to neutralize 99% of WT-rAAV6 vectors, whereas less than 5% of capsid-optimized rAAV6 vectors were neutralized at the same concentration. Thus, the capsid-optimized rAAV6 vectors were used in the following experiments to deliver exogenous genes into hematopoietic cells. We further found that rAAV6 vectors led to a ~10% transduction efficiency in the primary CD34+ HSCs and CD4+ T cells at an MOI of 10,000 vgs/cell (Figure 1C). 

### 3.2. In-Cis EMCV IRES Inhibited Transgene Expression in Hematopoietic Cells

**Figure 2:**
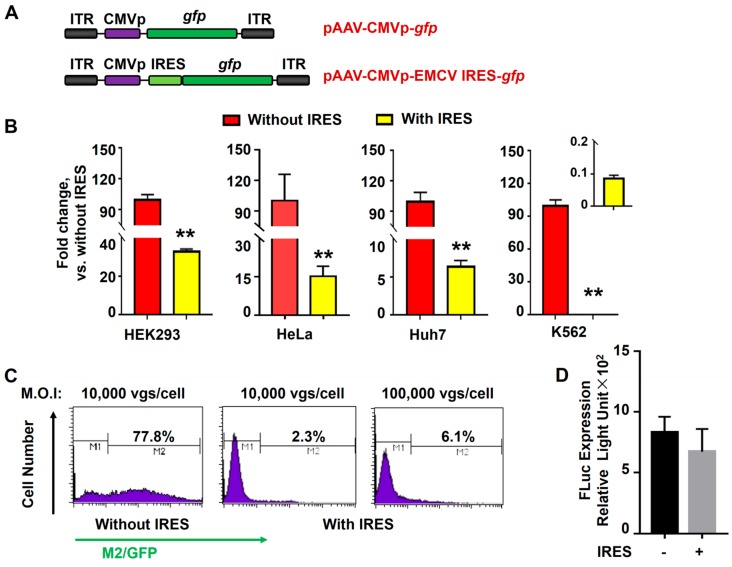
In-cis encephalomyocarditis virus (EMCV) internal ribosome entry site (IRES) inhibited the expression of transgene in K562 cells. (**A**) Diagram of the rAAV6 vector genomes. (**B**) HEK293, HeLa, Huh7, and K562 cells were transduced with rAAV6-CMVp-*gfp* or rAAV6-CMVp-EMCV IRES-*gfp* at 10,000 vgs/cell. Transgene expression was detected by fluorescence microscopy at 72 hours post-transduction. (**C**) Flow cytometry analysis of GFP-positive cell number in K562 cells transduced with rAAV6 vectors at the indicated MOI. Transgene expression was detected by flow cytometry at 72 hours post-transduction. (**D**) K562 cells were transduced with rAAV6-CMVp-*fluc* at 10,000 vgs/cell and coinfected with either rAAV6-CMVp-*gfp* or rAAV6-CMVp-EMCV IRES-*gfp* at 10,000 vgs/cell. The expression of firefly luciferase was measured at 72 hours post-transduction.

To investigate EMCV IRES-mediated transgene expression, we constructed pAAV-CMVp-*gfp* and pAAV-CMVp-EMCV IRES-*gfp* (Figure 2A). Both vectors were used to transduce various cell lines, including HEK293, HeLa, Huh7, and K562. As shown in Figure 2B, the EMCV IRES-containing genomes led to ~30%, ~15%, and ~6% efficiency in HEK293, HeLa, and Huh7 cells, respectively, compared to their counterparts without the EMCV IRES. Notably, a complete loss of transgene expression was observed when attempting to deliver EMCV IRES-containing genomes to K562 cells. The EMCV IRES-containing vector dose was further increased from 10,000 vgs/cell to 100,000 vgs/cell, whereas the GFP expression efficiency was enhanced from only 2.3% to 6.1% (Figure 2C). Furthermore, we also found that the inhibitory effect of EMCV IRES was cis-acting instead of trans-acting (Figure 2D).

**Figure 3:**
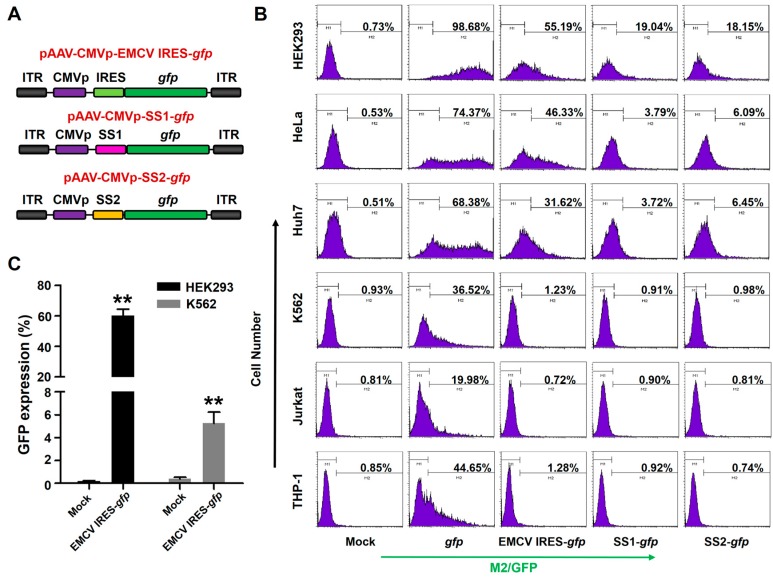
The effect of EMCV IRES on various cell types. (**A**) Diagram of the rAAV6 vector genomes. (**B**) Indicated cells were transduced with indicated rAAV6 vectors at 10,000 vgs/cell. Transgene expression was detected by flow cytometry at 72 hours post-transduction. (**C**) HEK293 and K562 cells were infected with LV-CMVp-EMCV IRES-*gfp* using lentiviral supernatant. Transgene expression was detected by flow cytometry at 7 days post-infection.

Next, we constructed two additional pAAV vectors with the equilong “stuffer sequence” (SS) as controls, which were denoted as pAAV-CMVp-SS1-*gfp* and pAAV-CMVp-SS2-*gfp* (Figure 3A). As shown in Figure 3B, the increased distance between the promoter and ORF significantly decreased GFP expression in HEK293 (SS1: 19.04%, SS2: 18.15% vs. 98.68%), HeLa (SS1: 3.79%, SS2: 6.09% vs. 74.37%), Huh7 (SS1: 3.72%, SS2: 6.45% vs. 68.38%), K562 (SS1: 0.91%, SS2: 0.98% vs. 36.52%), Jurkat (SS1: 0.90%, SS2: 0.81% vs. 19.98%) and THP-1 (SS1: 0.92%, SS2: 0.74% vs. 44.65%) cells. Interestingly, the EMCV IRES element rescued the transgene expression only in non-hematopoietic cells but not in hematopoietic cells. This indicated that the inhibitory effect of EMCV IRES is hematopoietic-specific. Furthermore, we investigated transgene expression when EMCV IRES-*gfp* was integrated in the host genome by using a lentiviral system. As shown in Figure 3C, the GFP expression in HEK293 and K562 cells was 60.29 ± 7.40% and 5.24 ± 1.69%, respectively. These results suggested that the EMCV IRES element that is imbedded in the host genome failed to completely shut down the transgene expression.

### 3.3. EMCV IRES Had a Similar Inhibitory Effect on the Double-Transgene Vector in Hematopoietic Cells

**Figure 4:**
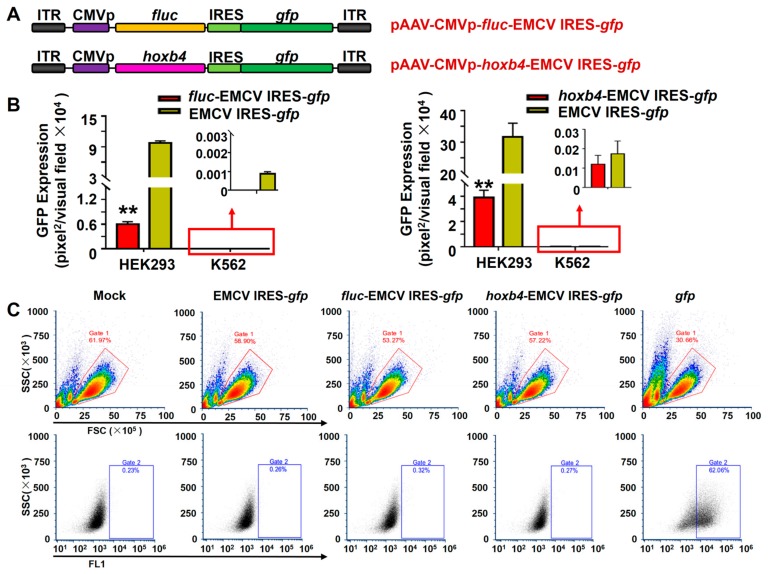
EMCV-IRES inhibited the expression of downstream transgene in hematopoietic cells. (**A**) Diagram of the rAAV6 vector genomes. (**B**) HEK293 cells and K562 cells were transduced with indicated rAAV6 vectors at 10,000 vgs/cell. Transgene expression was detected by fluorescence microscopy at 72 hours post-transduction. (**C**) Primary human CD34+ HSCs were transduced with indicated rAAV6 vectors at 10,000 vgs/cell. Transgene expression was detected by flow cytometry at 72 hours post-transduction.

We next constructed pAAV-CMVp-*fluc*-EMCV IRES-*gfp* and pAAV-CMVp-*hoxb4*-EMCV IRES-*gfp* vectors (Figure 4A). The GFP expression from these vectors and that from the pAAV-EMCV IRES-*gfp* vectors was determined side by side in HEK293 and K562 cells. Consistent with previous reports [15,35], the presence of an upstream transgene reduced the transgene expression of EMCV IRES-*gfp* gene. However, no GFP expression could be detected in K562 cells (Figure 4B) and primary human CD34+ HSCs (Figure 4C).

### 3.4. Potential Mechanism of EMCV IRES’s Inhibitory Effect in Hematopoietic Cells

**Figure 5:**
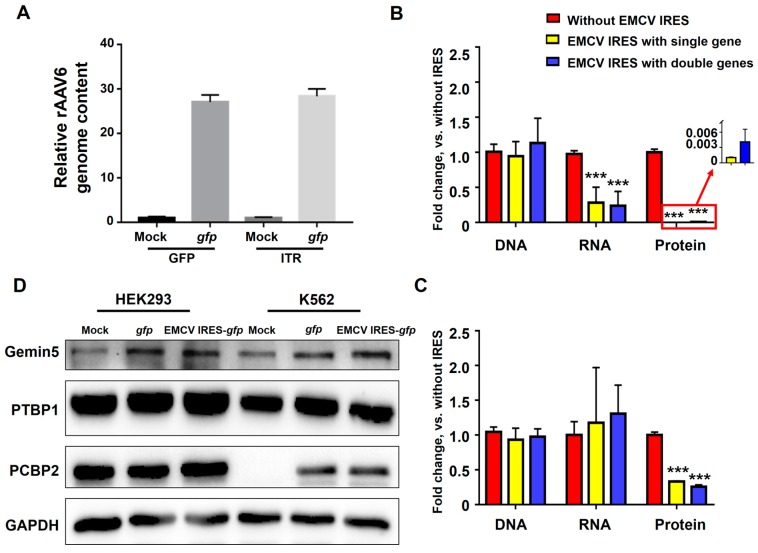
EMCV IRES inhibited the expression of transgene on the translational level. (**A**) HEK293 cells were transduced with rAAV6-CMVp-*gfp* at 10,000 vgs/cell. The relative rAAV6 genome content was detected by qPCR using GFP primers and ITR primers. (**B**) K562 and (**C**) HEK293 cells were transduced with rAAV6-CMVp-*gfp* and rAAV6-CMVp-EMCV IRES-*gfp* at 10,000 vgs/cell. Total DNA and RNA were isolated at 4 days post-transduction for qPCR. Transgene expression was detected by fluorescence microscopy at 72 hours post-transduction. **(D)** Western blot of total cell extracts (lysate) from HEK293 cells and K562 cells after rAAV6-CMVp-*gfp* or rAAV6-CMVp-EMCV IRES-*gfp* infection for Gemin5, PTBP1, and PCBP2 expression.

The relative rAAV6 genome contents were compared by using GFP primers and ITR primers. They showed a similar trend in qPCR assay, as shown in Figure 5A. Therefore, GFP primers were used in subsequent experiments. Next, we examined the GFP content at the genome, transcriptional, and translational levels in both HEK293 and K562 cells. Expression vectors harboring an EMCV IRES element showed similar GFP content to that of their counterparts without the IRES element at the mRNA level, as well as a three- to four-fold decrease of the protein level in HEK293 cells (Figure 5B). In contrast, a reduction of nearly 1,000-fold was observed at the level of translational product in K562 cells, with only a three-fold decrease in the mRNA level (Figure 5C). These results indicated that the inhibitory effect of EMCV IRES occurred during mRNA–protein transition. The differential expressions of key IRES binding proteins [36] including Gemin5, PTBP1, and PCBP2 were also analyzed between the HEK293 and K562 cells (Figure 5D). We found that PTBP1 was lower in the K562 cells than that in the HEK293 cells. PCBP2 expression was not detected in the mock group of K562 cells. These results suggest that PTBP1 and PCBP2 may be involved in the closure of EMCV IRES-mediated downstream gene expression in hematopoietic cells. In addition, methylation status of the CMV promoter in rAAV6 genomes was detected by bisulfite sequencing. Almost no methylation was found in the CMV promoter with or without the EMCV IRES element (data not shown) [37]. 

We next constructed five EMCV IRES-harboring vectors at different positions to evaluate whether GFP expression is dependent on the IRES position (Table 1). We observed that the GFP expression was 0.90 ± 0.66% of positive cells, which was extremely inhibited only when the IRES element was located between a promoter and the transgene. This indicated that the inhibition of transgene expression by IRES-harboring vectors was dependent on the IRES position in hematopoietic cells.

### 3.5. Comparison of Various Strategies to Express Dual Proteins in Hematopoietic Cells

**Figure 6:**
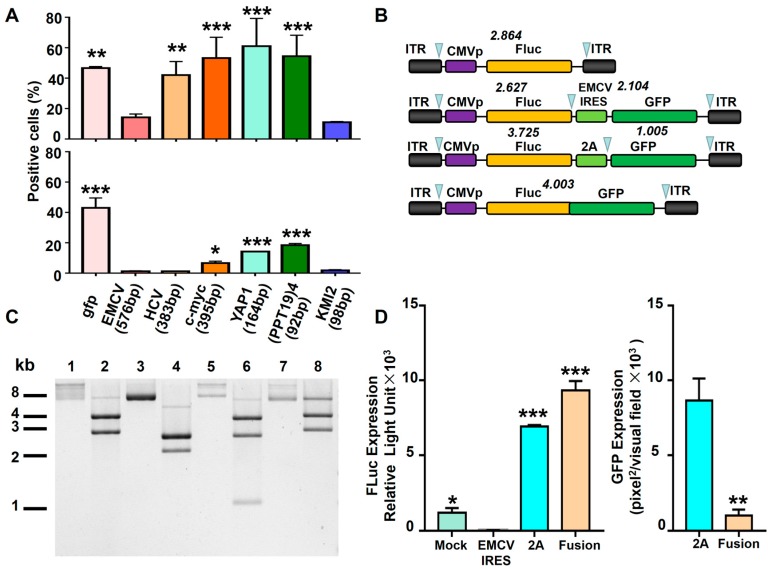
The transgene expression from various bicistronic rAAV6 vectors in hematopoietic cells. (**A**) HEK293 (upper) and K562 (lower) cells were transduced with rAAV6-CMVp-IRES-*gfp* at 10,000 vgs/cell. The six different IRES elements are described in the manuscript. Transgene expression was detected by flow cytometry at 72 hours post-transduction. (**B**) Diagram of the rAAV6 vector genomes. (**C**) Enzyme digestion of plasmids in (B). Lines 1, 3, 5, and 7 were undigested plasmids. Lines 2, 4, 6, and 8 were plasmids digested by SmaI. SmaI-pAAV-CMVp-*fluc* (11, 11, 2864, 4095bp); SmaI-pAAV-CMVp-*fluc*-EMCV IRES-*gfp* (11, 11, 2104, 2627, 2681bp); SmaI-pAAV-CMVp-*fluc*-2A-*gfp* (11, 11, 1005, 2651, 3725bp); SmaI-pAAV-CMVp-*fluc*-*gfp* (11, 11, 3007, 4003bp). (**D**) The expression of Fluc and GFP from (B) was detected at 72 hours post-transduction.

According to the above results, we found that EMCV IRES-containing bicistronic vectors almost could not express the target protein in K562 cells in most cases. To realize dual protein expression in hematopoietic cells with high efficiency, we investigated five additional IRESs that originated from viruses (HCV IRES), cells (c-myc IRES; YAP1 IRES), or artificial synthesis ((PPT19)4 IRES; KMI2 IRES). As indicated in Figure 6A, not all types of IRES could shut down GFP expression in K562 cells. Among them, c-myc IRES, YAP1 IRES, and (PPT19)4 IRES had the ability to mediate GFP expression. Moreover, we used another two approaches to design vectors: the intergenic insertion of a viral self-cleaving 2A peptide sequence and fusion gene (Figure 6B,C). The results demonstrate that the Fluc signal intensities of the pAAV-CMVp-*fluc*-2A-*gfp* group and pAAV-CMVp-*fluc*-*gfp* group were remarkably higher than that of the pAAV-CMVp-*fluc*-EMCV IRES-*gfp* group. This suggests that the 2A peptide and fusion gene methods are more appropriate for the transgene expression of bicistronic vectors in hematopoietic cells (Figure 6D).

## 4. Discussion

The low gene transduction efficiency of hematopoietic cells has always been a restraining factor for gene therapy in treating hematopoietic diseases [38]. Thus, there is an urgent need to develop a novel transgene method to efficiently increase exogenous gene transduction with low or tolerable adverse effects on blood-disease patients. To this end, we used rAAV vectors, which have the advantages of low immunogenicity and being non-pathogenic [39]. We compared rAAV6 and rAAV-DJ vectors with other commonly used non-viral gene transfer systems. As expected, rAAV6 vectors exerted the best transgene expression efficiency among these approaches in human hematopoietic cells. Similar findings were obtained previously, in which rAAV6 had considerably high tropism for human HSPCs [26,27,40]. It was also reported that rAAV6 vectors have lower efficiency in mouse HSPCs [26]. We obtained similar results that rAAV6 vectors inefficiently transduced primary rat peripheral blood lymphocytes (*gfp*: 0.78% vs. EMCV IRES *gfp*: 0.73%) and peripheral blood mononuclear cells (*gfp*: 0.49% vs. EMCV IRES *gfp*: 0.62%). Thus, mouse or rat cells cannot be used to study the effect of IRES elements in this study.

We concluded that the IRES element derived from EMCV mediates the shut-down of transgene expression and thus is not an ideal option to introduce dual transgenes into the hematopoietic cells. A major concern is that the decreased transgene expression was due to the increased distance between the promoter and ORF. To rule out this possibility, we constructed single-gene expression vectors with or without EMCV IRES, as well as the equilong SS as controls. Importantly, the EMCV IRES element could rescue transgene expression in non-hematopoietic cells, while it completely lost this function in hematopoietic cells. It is also worth mentioning that two pairs of IRES elements in our study have similar lengths, c-myc IRES (395 bp) vs. HCV IRES (383 bp) and (PPT19)4 IRES (92 bp) vs. KMI2 IRES (98 bp). We observed that c-myc IRES and (PPT19)4 IRES had a function in K562 cells (Figure 6A), indicating that distance is not a crucial factor for the elimination of transgene expression. Taken together, we reasoned that EMCV IRES-mediated transgene expression closure is not simply due to the distance.

The detailed molecular mechanism still warrants further exploration. Through the lentiviral infection system, we found that transgene expression failed to be completely shut down when EMCV IRES-*gfp* was imbedded in the host genome. This may be attributed to the different cellular mechanisms between AAV- and LV-mediated transgene expressions. In addition, the EMCV IRES shut-down of transgene expression seemed to happen during mRNA–protein transition. The role of EMCV IRES’s secondary structure, which forms steric hindrance, is unknown. Furthermore, our results suggested the involvement of cellular factors PTBP1 and PCBP2 in hematopoietic cells. Although we could not fully interpret this biological phenomenon as of yet, it strongly motivates us to seek the underlying mechanism of EMCV IRES-mediated transgene expression in hematopoietic cells. Currently, RNA pull down and RNA immunoprecipitation (RIP) assays are underway in our laboratory.

Over the past decades, many gene co-expression strategies have been reported in gene therapy experiments, such as 2A peptide, multiple promoter, fusion protein, and reinitiation methods [41]. The limitation of EMCV IRES element sparked us to identify suitable systems in hematopoietic cells. Thus, two further vector-construction approaches were employed: the viral self-cleaving 2A peptide linker [42] and fusion protein [43]. Importantly, we revealed that these two bicistronic vectors showed higher transgene efficiency in hematopoietic cells. The observations warrant further study, as these methods enable hematopoietic cells to express specific transgenes, which may be important for gene therapy applications.

In summary, although the rAAV6 vector exhibited outstanding transduction efficiency in hematopoietic cells, the presence of the EMCV IRES element in the viral vector genome almost completely shut down transgene expression. To our knowledge, our findings are the first to demonstrate that IRES elements dramatically suppress transgene expression in hematopoietic cells. Although negative results were presented, our study is still interesting for researchers in the field, especially considering the current importance of the rAAV vector in hematopoietic gene delivery and gene editing.

## Figures and Tables

**Table 1:** The GFP expression of capsid-optimized rAAV6 vectors with EMCV IRES at various positions in HEK293 and K562.

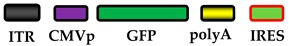	HEK293 Positive Cells (%)	K562 Positive Cells (%)
**Mock**	1.15 ± 0.27	1.21 ± 0.41
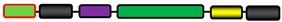	53.77 ± 12.05	53.59 ± 7.09
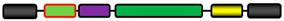	64.94 ± 17.74	44.18 ± 7.31
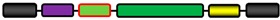	11.71 ± 2.81	0.90 ± 0.66
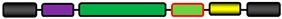	69.21 ± 12.17	44.53 ± 6.21
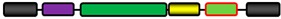	70.95 ± 9.43	46.00 ± 12.36
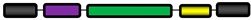	69.95 ± 13.18	61.27 ± 16.82
